# Development of the reproduction number from coronavirus SARS-CoV-2 case data in Germany and implications for political measures

**DOI:** 10.1186/s12916-020-01884-4

**Published:** 2021-01-28

**Authors:** Sahamoddin Khailaie, Tanmay Mitra, Arnab Bandyopadhyay, Marta Schips, Pietro Mascheroni, Patrizio Vanella, Berit Lange, Sebastian C. Binder, Michael Meyer-Hermann

**Affiliations:** 1 Department of Systems Immunology and Braunschweig Integrated Centre of Systems Biology (BRICS), Helmholtz Centre for Infection Research, Rebenring 56, Braunschweig, 38106 Germany; 2 Department of Epidemiology, Helmholtz Centre for Infection Research (HZI), Inhoffenstr. 7, Braunschweig, 38124 Germany; 3 Hannover Biomedical Research School (HBRS), Carl-Neuberg-Str. 1, Hannover, 30625 Germany; 4 Chair of Empirical Methods in Social Science and Demography, University of Rostock, Ulmenstr. 69, Rostock, 18057 Germany; 5 German Center for Infection Research (DZIF), Inhoffenstraße 7, Braunschweig, 38124 Germany; 6 Institute for Biochemistry, Biotechnology and Bioinformatics, Technische Universität Braunschweig, Braunschweig, Germany; 7 Cluster of Excellence RESIST (EXC 2155), Hannover Medical School, Carl-Neuberg-Straße 1, Hannover, 30625 Germany

**Keywords:** SARS-CoV-2, COVID-19, Epidemiology, Modeling, Non-pharmaceutical interventions, Reproduction number, Healthcare usage

## Abstract

**Background:**

SARS-CoV-2 has induced a worldwide pandemic and subsequent non-pharmaceutical interventions (NPIs) to control the spread of the virus. As in many countries, the SARS-CoV-2 pandemic in Germany has led to a consecutive roll-out of different NPIs. As these NPIs have (largely unknown) adverse effects, targeting them precisely and monitoring their effectiveness are essential. We developed a compartmental infection dynamics model with specific features of SARS-CoV-2 that allows daily estimation of a time-varying reproduction number and published this information openly since the beginning of April 2020. Here, we present the transmission dynamics in Germany over time to understand the effect of NPIs and allow adaptive forecasts of the epidemic progression.

**Methods:**

We used a data-driven estimation of the evolution of the reproduction number for viral spreading in Germany as well as in all its federal states using our model. Using parameter estimates from literature and, alternatively, with parameters derived from a fit to the initial phase of COVID-19 spread in different regions of Italy, the model was optimized to fit data from the Robert Koch Institute.

**Results:**

The time-varying reproduction number (*R*_t_) in Germany decreased to <1 in early April 2020, 2–3 weeks after the implementation of NPIs. Partial release of NPIs both nationally and on federal state level correlated with moderate increases in *R*_t_ until August 2020. Implications of state-specific *R*_t_ on other states and on national level are characterized. Retrospective evaluation of the model shows excellent agreement with the data and usage of inpatient facilities well within the healthcare limit. While short-term predictions may work for a few weeks, long-term projections are complicated by unpredictable structural changes.

**Conclusions:**

The estimated fraction of immunized population by August 2020 warns of a renewed outbreak upon release of measures. A low detection rate prolongs the delay reaching a low case incidence number upon release, showing the importance of an effective testing-quarantine strategy. We show that real-time monitoring of transmission dynamics is important to evaluate the extent of the outbreak, short-term projections for the burden on the healthcare system, and their response to policy changes.

**Supplementary Information:**

The online version contains supplementary material available at (10.1186/s12916-020-01884-4).

## Background

The outbreak of the novel coronavirus SARS-CoV-2 (CoV/COVID-19) in China has induced a worldwide pandemic. The comparably high lethality in the elderly population and in patients with comorbidities [1, 2], together with a widely absent immunization of the population against the novel virus as well as the limited health system capacity estimated to become overwhelmed by an unlimited virus spreading [3], led to non-pharmaceutical interventions (NPIs) to reduce virus transmission mostly by reducing inter-individual contacts. The aim of these measures was to achieve at least a delay of viral spreading, allowing the healthcare system to extend its capacities and to treat less patients per time or, ideally, achieve a complete stop of viral spreading. The NPIs installed in Germany have been effective in containing viral dissemination [4]. Hence, in the light of economic damage incurred by restrictions [5], a gradual release of NPIs was decided with moderate effects on virus transmission. However, the length of the serial interval, which is in the range of 4–7.5 days (mean values) for CoV [6–8], and an inevitable delay in testing and reporting imply that any sudden outbreaks may be recognized too late and careful continuous monitoring of the infection dynamics on a regional basis is required. Thus, current political decisions need foundational information about current infection dynamics and their response to changes in NPIs such as partial release of contact restrictions or school openings, ideally on a regional basis. In fact, a declining or stable number of daily reported cases despite releasing measures can be misleading if the trend of the achieved reproduction number, the delay between changes in the infection dynamics, and their manifestations in reported case numbers are not taken into account. Furthermore, the high variance of the locally reported new cases adds to this uncertainty. Thus, it is extremely important to construct a model that not only captures the disease dynamics but also has the potential to provide information on the trend of the outbreak by considering the time-dependence of the reproduction number for COVID-19. The situation in Europe was recently analyzed [7]. Here, a systematic analysis of the development of the reproduction number over the time period of the COVID-19 outbreak in Germany and in all federal states of Germany is provided.

A second level of information necessary for political decisions on NPIs is the prospective development of the outbreak under different scenarios. A too early release of NPIs risks to abandon the current level of containment and to initiate a new wave of viral spreading [9]. A too long application of NPIs carries the risk of collateral damage and imposes a strong economic burden [5]. In view of the achieved reproduction number in Germany and its federal states by April 2020, a partial release of NPIs was decided, including partial school re-openings and resumption of catering and hotel business under certain restrictions, including mask obligations in public. The effects of such re-openings are hard to predict and require careful monitoring of local factors governing the infection dynamics and their implications for forecasting the immediate future development of the pandemic.

Whereas existing data-based simple algorithms (e.g., [10]) stand useful for estimating the time-varying reproduction number of an ongoing outbreak for many infectious diseases (e.g., measles, H1N1 swine flu, polio) using symptom onset data and less parameters (e.g., serial interval only), they, in general, are designed considering detection of all cases and are not suitable when the proportion of detected cases may change over time. Furthermore, the absence of reliable symptom onset data and heterogeneity of an effective infectious period among asymptomatic, pre-symptomatic, and symptomatic individuals demand for an alternative method to estimate the time-varying reproduction number by adjusting for such specific features of COVID-19. We have developed an ordinary differential equation (ODE)-based compartmental model specific for COVID-19 transmission dynamics and disease progression, and used it for quantitative evaluation of the time-varying reproduction number under the influence of NPIs in Germany and its federal states. In addition, our contribution retrospectively infers the usage of the healthcare system in Germany and offers short-term predictions based on current developments in terms of infections, number of non-critical hospital beds, and critical/intensive care units (ICUs) needed to treat patients with severe disease progression, as well as fatalities. This analysis provides additional information on when and how strongly to react to potential infection waves in order to avoid unacceptably high mortality and morbidity as well as excessive demands on the healthcare system. As a state-specific estimation of the reproduction numbers and a prospective estimation of the outbreak need to be up-to-date for the purpose of closely monitoring effects of policy changes, we provide daily updates of our analysis results online [11].

## Methods

**Fig. 1:**
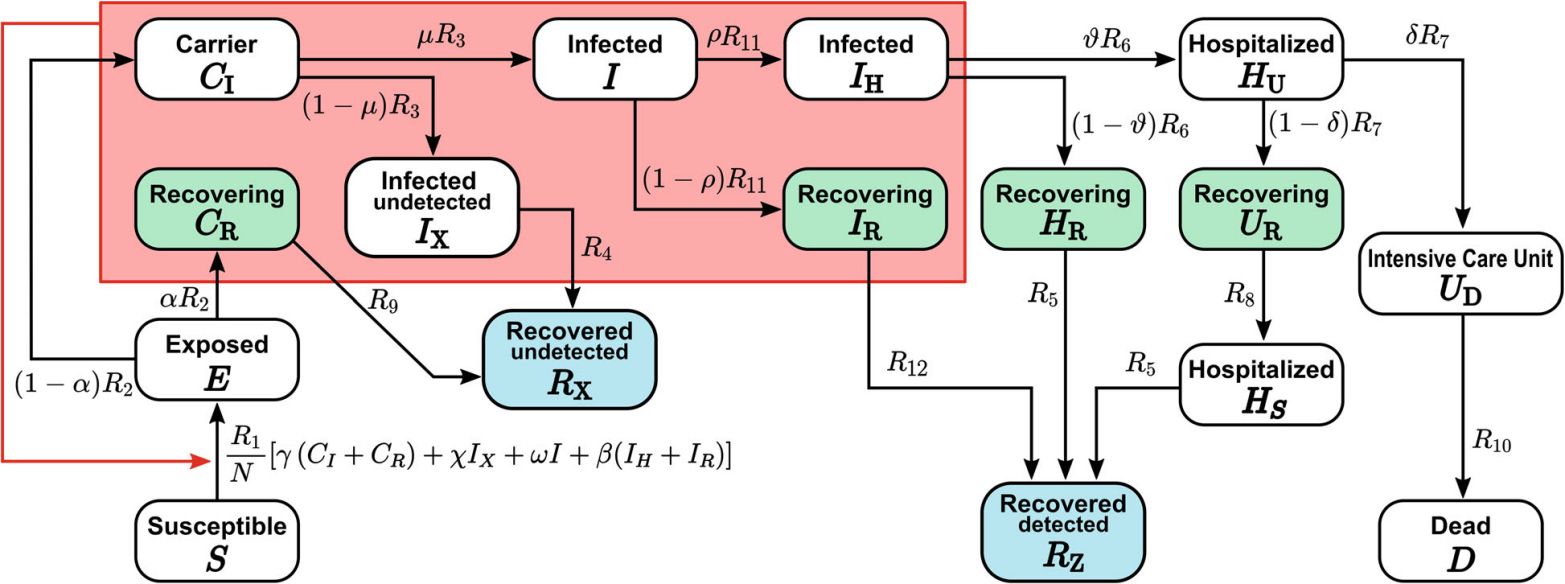
The scheme of the SECIR model, which distinguishes susceptible (*S*) individuals without immune memory of CoV, exposed (*E*) who already carry the virus but are not yet infectious to others, carriers (*C*_*I*,*R*_) who carry the virus and are infectious to others but do not show symptoms, infected (*I*,*I*_*H*,*R*,*X*_) who carry the virus with symptoms and are infectious to others, hospitalized (*H*_*U*,*R*,*S*_) who experience a severe development of the disease, patients transferred to intensive care unit (*U*_*R*,*D*_), dead (*D*), and recovered (*R*_*Z*,*X*_) who acquired immune memory and cannot be infected again. Recovery happens from each of the states *C*_*R*_,*I*_*X*_,*I*_*R*_,*H*_*R*_,*U*_*R*_. See Table 1 for parameter values

The implemented SECIR (Susceptible-Exposed-Carrier-Infected-Recovered) model is a deterministic ODE model adapted to the specific properties of SARS-CoV-2 viral infections. It distinguishes healthy individuals without immune memory of COVID-19 (*S*), infected individuals without symptoms but not yet infectious (*E*), infected individuals without symptoms who are infectious (pre-symptomatic (*C*_*I*_) and asymptomatic (*C*_*R*_) carriers), infected symptomatic individuals who are not yet detected (*I*), and detected (*I*_*H*,*R*_) and undetected (*I*_*X*_) symptomatic patients. Further, compartments for hospitalization in non-critical (*H*_*U*,*R*,*S*_) and critical/intensive care units (*U*_*D*,*R*_) were introduced to monitor the load on the healthcare system. Detected patients recover from different states of the disease (*R*_*Z*_) or die (*D*). Undetected individuals who went through the infection and recovered are also taken into account (*R*_*X*_). The quantities are defined, and the model is summarized in Fig. 1 with parameters in Table 1. The model equations read

1 $$\begin{array}{*{20}l} {}\frac{dS}{dt} &\,=\, -R_{1}\!(t)\! \frac{\left(\gamma (C_{I}+C_{R})+\chi I_{X}+\omega I + \beta (I_{H}+I_{R})\right)}{N}S \end{array} $$

**Table 1:** Parameter sets of the SECIR model: determination of the mean values (and ranges where applicable, e.g., *𝜗*, *δ*) for literature-based parameter set was based on the interpretation of the values given in the references and is discussed in the supporting information (see Parameter description [12–20]). The Italy-based parameter set was determined by fitting the data for different regions of Italy and providing minimum and maximum over the analyzed regions. While *α* and *R*_7_ were indeed kept fixed for Italy, other parameter ranges are the result of the fitting itself. Results are shown up to three decimal points

Parameter(s)	References	Parameter set from literature		Parameter set from fitting Italy data	
		Mean	Variation	Min	Max
***R***_***1***_	Varied, fitted (see the “Methods” section)				
***R***_***2***_	[8, 21, 22]	$\frac {1}{3.2}$	20%-around mean	0.219	0.406
***R***_***3***_	1/R _3_ = 5.2 – 1/ *R*_2_ ; mean incubation period is 5.2 days [8]				
***R***_***4***_	[23–25]	$\frac {1}{7}$	20%-around mean	0.100	0.186
***R***_***5***_	[26, 27]	$\frac {1}{8}$	20%-around mean	0.088	0.088
***R***_***6***_	1/R _6_ = *X*– 1/ *R*_11_; *X* = 4.25 (literature [28]), 5 (Italy fitting); time to hospitalization since symptom is *X* days				
***R***_***7***_	[27, 28]	$\frac {1}{4.25}$	20%-around mean	1	1
***R***_***8***_	[26]	$\frac {1}{9}$	20%-around mean	0.144	0.144
***R***_***9***_	${\frac {1}{R_{9}}=\ \ \frac {1}{R_{3}}+\left (0.5\ \times \ \frac {1}{R_{4}}\right)}$				
***R***_***10***_	[28, 29]	$\frac {1}{7.43}$	20%-around mean	0.082	0.113
***R***_***11***_	$\frac {1}{R_{11}}=\ 3.7$ days [30]; fixed				
***R***_***12***_	$\frac {1}{R_{12}}=\ \ \frac {1}{R_{4}}- \frac {1}{R_{11}}$				
***α***	[31, 32]	NA	Fixed to 0.22 except Additional file 1: Figure S4	0.425	0.425
***β***	Assumed	NA	0.05–0.25	0.050	0.050
***ρ***	Varied, fitted (see the “Methods” section)				
***𝜗***	[33, 34]	NA	0.42–0.53	0.157	0.311
***δ***	[33, 34]	NA	0.42–0.53	0.300	0.900
***μ***	Varied (see the “Methods” section); calculations from Italy data (see Additional file 1: Additional details of Italy fitting [35, 36]) yield in an average (drawn from the analyzed regions) *μ* = 0.13 with *α* = 0.425 [31]				
***γ;χ;ω***	Assumed; fixed to 1

2 $$\begin{array}{*{20}l} {}\frac{dE}{dt} &\,=\, R_{1}\!(t) \frac{\left(\gamma\! (C_{I}\,+\,C_{R})\,+\,\chi I_{X}\,+\,\omega I + \beta (I_{H}+I_{R})\right)}{N}S-R_{2}E \end{array} $$

3 $$\begin{array}{*{20}l} {}\frac{dC_{I}}{dt} &= {\left(1-\alpha \right)R}_{2}E-R_{3}C_{I} \end{array} $$

4 $$\begin{array}{*{20}l} {}\frac{dC_{R}}{dt} &= \alpha R_{2}E\ -{R_{9}C}_{R} \end{array} $$

5 $$\begin{array}{*{20}l} {}\frac{dI}{dt} &= \mu {R_{3}C}_{I}-R_{11}I \end{array} $$

6 $$\begin{array}{*{20}l} {}\frac{dI_{H}}{dt} &= {\rho(t) R_{11}I}-R_{6}I_{H} \end{array} $$

7 $$\begin{array}{*{20}l} {}\frac{dI_{R}}{dt} &= \left(1-\rho(t) \right)R_{11}I-R_{12}I_{R} \end{array} $$

8 $$\begin{array}{*{20}l} {}\frac{dI_{X}}{dt} &= \left(1-\mu \right)R_{3}C_{I}-R_{4}I_{X} \end{array} $$

9 $$\begin{array}{*{20}l} {}\frac{dH_{U}}{dt} &= \vartheta R_{6}I_{H}-R_{7}H_{U} \end{array} $$

10 $$\begin{array}{*{20}l} {}\frac{dH_{R}}{dt} &= \left(1-\vartheta \right)R_{6}I_{H}-R_{5}H_{R} \end{array} $$

11 $$\begin{array}{*{20}l} {}\frac{dH_{S}}{dt} &= R_{8}U_{R}-\ R_{5}H_{S} \end{array} $$

12 $$\begin{array}{*{20}l} {}\frac{dU_{D}}{dt} &= \delta R_{7}H_{U}-R_{10}U_{D} \end{array} $$

13 $$\begin{array}{*{20}l} \frac{dU_{R}}{dt} &= \left(1-\delta \right)R_{7}H_{U}-R_{8}U_{R} \end{array} $$

14 $$\begin{array}{*{20}l} \frac{dR_{Z}}{dt} &= R_{12}I_{R}+\ R_{5}H_{R}+\ R_{5}H_{S} \end{array} $$

15 $$\begin{array}{*{20}l} \frac{dR_{X}}{dt} &= R_{9}C_{R}+\ R_{4}I_{X} \end{array} $$

16 $$\begin{array}{*{20}l} \frac{dD}{dt} &= R_{10}U_{D} \end{array} $$

The rates $R_{2,\dots,12}$ denote the inverse time of transition between the respective states and can be estimated from literature. Parameter *R*_1_ is fitted to the course of reported case numbers in a sliding time window and therefore is a time-varying parameter. Greek letters *α*,*μ*,*ρ*,*𝜗*, and *δ* denote fractions of individuals with a particular fate while other Greek letters, viz., *γ*,*χ*,*ω*, and *β*, reflect the intensity of interaction of corresponding infectious compartments with the susceptible population. The overall case fatality ratio (CFR = *ρ𝜗δ*) has a time-varying component modeled with a logistic function

17 $$\begin{array}{*{20}l} \text{CFR} (t) &= H - (H-L) \left(\frac{1}{1+e^{-k(t-t_{0})}} \right), \end{array} $$

where *t* corresponds to the day of the year starting from January 1, 2020, and *H*=0.139,*L*=0.007,*k*=0.145, and *t*_0_=87.3 (these values are for *μ*=0.2) are obtained from fitting the curve for cumulative deaths to obtain a time-dependent case fatality rate (CFR), which changed over the course of the epidemic in Germany. This is due to changing testing frequencies [37] and the shifting age structure of the infected over time [38], which we assume to predominantly reflect in a time-varying rate of hospitalization (*ρ*(t)).

The time-dependent *ρ*(*t*) is effectively incorporating the time-varying age distribution of the infected people in the course of the epidemic. An explicit representation of the age distribution [39] was not favored in view of many unknown parameters. Note that hospitalization occurs from a quarantined compartment in the model, thereby having relatively less influence on the *R*_t_ values. Hence, *ρ*(t) as resulted from CFR(t) in the country level was retained for estimating federal state-specific time-varying reproduction numbers. The demographic differences may be more important for the analysis of smaller districts; however, case numbers in smaller districts might not be sufficient for a proper discrimination of age groups.

### Parameterization

The model parameters are critical for the overall behavior of the model and for the quality of the predictions derived from it. For the sake of robustness of the results, we followed two different strategies on how to determine the model parameters. The development for time- varying reproduction numbers in Germany (Fig. 3a) was presented with the parameter sets derived from both strategies.

The first strategy was to derive the estimated values (see Table 1) of the model parameters for Germany based on the available literature (e.g., [26]). Some disease-specific quantities such as the incubation period and infectious period are considered to be independent of a specific country. Uncertainty in the values of the parameters was invoked either using a percentage variation (20% unless otherwise specified) around their estimated mean values or by sampling from an estimated range (e.g., *𝜗* and *δ*) (see Table 1 for details). The resulting ranges were subsequently used to determine the distribution of *R*_*t*_ values.

**Fig. 2:**
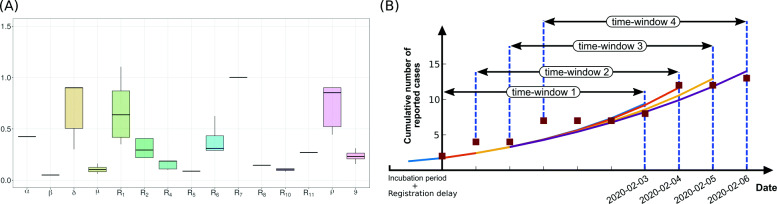
**a** Variability of parameters fitted to the number of reported, hospitalized, ICU, and dead cases in different regions of Italy. Table 1 recollects the parameter ranges. Note that the resulting ranges of *R*_1_ and *ρ* are not used for fitting case data of Germany. They are varied to optimize the model dynamics to case data in Germany (see the “Methods” section). **b** Scheme of the shifting time window and repeated fitting to the time course of the reported case data (shown for a window size of 7 days)

**Fig. 3:**
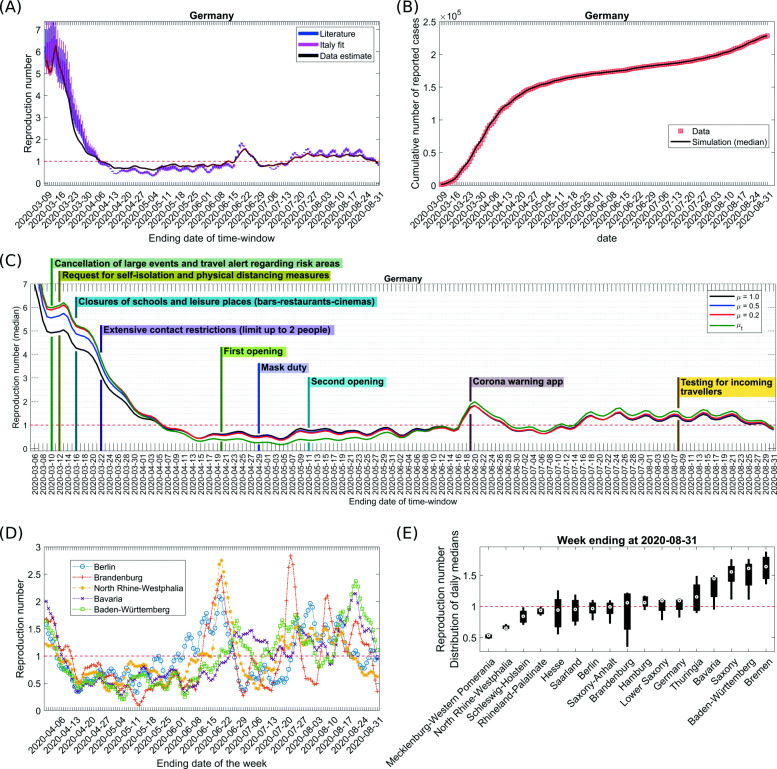
Data for Germany were fitted to the cumulative number of reported cases in a sliding time window with a size of 10 days. Parameters from Table 1 were used and the transmission rate *R*_1_ was fitted (see the “Methods” section). **a** Time-varying reproduction number *R*_*t*_ resulting from the fit. The parameter sets were randomly sampled within the ranges in Table 1, and upon refitting, this induced a variability of reported *R*_*t*_ values. The box plot shows the median, 25 and 75 percentiles, and the minimum and the maximum values. Both used parameter sets (literature-based with *μ*=0.2 and derived from Italy-fit) are compared to the *R*_*t*_ values calculated with the publicly available code from Imperial College (black curve) with a serial interval of 6.5 days having standard deviation of 0.62 days as used in [7]. **b** The median of fitting results in **a** with literature-based parameters is shown for the cumulative number of reported cases and compared with data from [41–43]; own calculation and design. **c** Same analysis based on the literature-based parameter set together with the timing of installing and releasing NPIs in Germany for *μ*=1,*μ*=0.5,*μ*=0.2, and time-varying *μ* (evaluated on the basis of mobility and testing data, see Additional file 1: Figure S1-S2). Only the median value is reported. **d** The same analysis as in **a** for federal states with *μ*=0.2. Results (only the median values) for Berlin, Brandenburg, Baden-Würtemberg, Bavaria, and North Rhine-Westphalia are shown for April 2020–August 2020. **e** The same analysis as in **a** was repeated for each federal state in Germany separately with *μ*=0.2 (see Additional file 1: Figure S3). Here, the *R*_*t*_ distribution resulted from the median *R*_*t*_ values past 1 week observed in each federal state of Germany is shown as box plot (see **a**). Federal states are sorted by median values of their *R*_*t*_ distribution. The horizontal line shows *R*_*t*_ = 1. **a**–**e** Each data point is a result of 100 randomly sampled parameter sets for a fixed *α*=0.22

In the second approach, we kept model parameters open in a broader range (see Additional file 1: Table 1 [27, 30, 31]) and fitted them to the cases reported in different regions of Italy until March 18, 2020, in a single stretch using our model assuming that the dynamics in this initial phase of the outbreak are not affected by the overwhelmed healthcare system. We optimized the model parameters to result in minimized error over this whole period. As the lockdown was announced on March 9, 2020, in Italy, an additional duration of 9 days (i.e., the sum of the incubation period of 5.2 days and a period of 3.7 days until clinical visit) was considered. Available data for cumulative infected, hospitalized, ICU, and deaths were fitted for Italy and for the regions where the first registered case was on February 28, 2020, or prior [40]. The diversity of resulting parameter values for the different regions in Italy (Fig. 2a) was used to derive a second range of the parameters to determine the distribution of *R*_*t*_ values (Fig. 3).

### Basic (*R*_0_) and time-varying (*R*_*t*_) reproduction number

The basic reproduction number *R*_0_ is defined as the expected number of secondary cases produced by a single infection in a completely susceptible population [44]. It can be calculated from the parameters of a compartmental model [45–48] after fitting the model to data for a given time period during the epidemic. While *R*_0_ provides valuable information on the viral dissemination dynamics in the absence of immunity and awareness of the epidemic, the dynamics of the epidemic over time will be heavily influenced by development of immunity in the population [49], policy changes to minimize infection risk [50], and individual behavior in response to public awareness of a disease [51]. Hence, a practically more useful quantity during an outbreak is the time-dependent reproduction number *R*_*t*_ describing the expected number of secondary cases per index case at a given time of the epidemic. This quantity has to be derived from incidence data over time and reflects the multifactorial impact of NPIs, behavior changes, seasonal effects, etc. on the dynamics of viral spread.

In epidemic models with multiple compartments, *R*_0_ can be derived with the next generation matrix method [47]. The compartments with infected individuals are divided into two contributions with respect to their dynamics: new infections entering the compartments and transfer of infected into and out of the compartment to other compartments. The Jacobian matrices of these two quantities *F* and *V* describe the generation of new infections and the transfer across compartments, respectively [52, 53]. The elements *G*_*ij*_ of *G*=*FV*^−1^ are related to the expected number of secondary infections in compartment *i* caused by a single infected individual of compartment *j*.

The reproduction number *R*_0_ for the present model is given by the dominant eigenvalue of *G*, i.e.:

18 $$ \begin{aligned} R_{0} &\,=\, R_{1} \frac{S_{0}}{N_{0}} \left[ \frac{\gamma \left(1 - \alpha \right)}{R_{3}} \,+\, \frac{\gamma \alpha}{R_{9}} \,+\, \frac{\chi \left(1 - \alpha \right) \left(1 - \mu \right)}{R_{4}} \,+\, \frac{\mu \omega \left(1 - \alpha \right)}{R_{11}} \right.\\ &\left.\qquad\quad + \frac{\beta \mu \left(1 - \alpha \right) \left(1 - \rho \right)}{R_{12}} + \frac{\beta \mu \rho \left(1 - \alpha \right) }{R_{6}} \right] \end{aligned}  $$

where *N*_0_ is the total population and *S*_0_ is the susceptible population, both at the start of the outbreak.

For our analysis, we use reported cases (*I*_*H*_+*I*_*R*_) in the first 4 days and impose the adjusted number of corresponding detectable infected cases (*N*_*i*_/[(1−*α*)*μ*], where *N*_*i*_ represents the number of reported cases at day *i*, *i*=1,2,3,4), as exposed individuals consecutively at an earlier time point given by the sum of one incubation period and the duration until clinical registration following symptom onset (1/*R*_2_+1/*R*_3_+1/*R*_11_=8.9 days). This assumption takes into account that the symptom onset from the first exposed individuals has not happened before the minimum duration of serial interval (around 4 days), and thus, the first reported cases shall represent independent sources of the virus rather than being the result of transmissions. Given the initial conditions and using the parameter sets in Table 1, the transmission parameter *R*_1_, which mostly contains information on the contact frequency and probability of transmission per contact and, thus, best reflects the individual behavior in the population with respect to social distancing and other measures to minimize the infection risk, is used to optimize the fit of the model dynamics to the observed case data.

In order to assess the impact of political measures and changes in the population response along with other dynamic but unrecognized variables (such as seasonality) onto the development of the time-varying reproduction number *R*_*t*_, the cumulative number of registered cases is used. The cumulative registered case number is compared to the sum of infected individuals and all subsequent states in the model, i.e., with *I*_*H*_+*I*_*R*_+*H*_*U*_+*H*_*R*_+*H*_*S*_+*U*_*R*_+*U*_*D*_+*D*+*R*_*Z*_. A time window of a width of 10 days is defined starting at the day of the first reported case (i) considering the time-difference between viral exposure and clinical registration of a case (which is around 9 days as per the model parameters) and (ii) assuming a delay of 1 day between the announcement of a new measure and changed personal behavior, the combination of which we use to define our default window size. For a window size of *WS* (our default *WS*=10 days), *R*_*t*_ shown on a particular date *D*_*t*_ depicts the reproduction number observed over a period from *D*_(*t*−*WS*)_ to *D*_*t*_. This allows to determine *R*(*t*_0_) in the first 10 days and to define the initial conditions for the first sliding time window (Fig. 2b). Then, in repeating cycles, the best *R*(*t*_*k*_) (with *k*=1...*N*) for each time window at the starting time *t*_*k*_ of the *k*th time window is determined by

19 $$ \begin{aligned} R(t_{k}) &\,=\, R_{1}(t_{k}) \frac{S(t_{k})}{N(t_{k})} \!\left[\! \frac{\gamma \left(1 - \alpha \right)}{R_{3}} + \frac{\gamma \alpha}{R_{9}} + \frac{\chi \left(1 - \alpha \right) \left(1 - \mu \right)}{R_{4}}\right.\\ &\qquad\qquad\qquad\,+\, \frac{\mu \omega \left(1 - \alpha \right)}{R_{11}} \,+\, \frac{\beta \mu \left(1 - \alpha \right) \left(1 - \rho(t_{k}) \right)}{R_{12}} \\ &\qquad\qquad\qquad\left.\!+ \frac{\beta \mu \rho(t_{k}) \left(1 - \alpha \right) }{R_{6}} \right] \end{aligned}  $$

where *ρ*(*t*_*k*_) denotes the average value of the time-varying hospitalization rate in the *k*th time window. A new set of initial conditions is defined a day later, including the reduced fraction of susceptible individuals *S*(*t*_*k*_)/*N*(*t*_*k*_), with *S*(*t*_*k*_) and *N*(*t*_*k*_) the values of susceptible and total population at the starting time *t*_*k*_ of the *k*th time window. Note that fatal cases reduce the total population. *R*_1_(*t*_*k*_) is determined by fitting to the data in this time window. In cycles, the time window is shifted 1 day later. The series of *R*_*t*_ values for each of the sliding time windows at time *t*_*k*_ is reported at the final date of the time window.

For the prospective study, the state of the model at the last time of *R*_*t*_ evaluation is kept and used as initial condition for the model.

To assess the impact of different scenarios, a set of constant reproduction numbers (*R*) was imposed based on the history of the epidemic to mimic release, maintenance, or intensification of NPIs (see the “Results” section). The cumulative number of infected individuals and the number of occupied ICUs, hospital beds, and deaths are reported. More observables are found at [11].

The distribution of observables and *R*_*t*_ values is generated by reiteration of the analysis under varying model parameters randomly drawn from a uniform distribution within the range provided in Table 1. The box plots in the figures show median, 25 and 75 percentiles, and minimum and maximum values from these analyses.

## Results

Based on the classical models of infection epidemics [52], we developed a mathematical model particularly adapted to the specificities of the COVID-19 outbreak (SECIR model, Fig. 1). For the evaluation of effects of NPIs and behavioral changes on viral spreading, a time-varying reproduction number *R*_*t*_ has to be estimated [10]. We opted for a shifting time window of 10 days (in Fig. 2b, a scheme with a window of 7 days is shown) in each of which *R*_*t*_ is determined, and developed an automatized algorithm for the fast analysis of the current *R*_*t*_ (see the “Methods” section, Eq. 19). Importantly, each time window is not analyzed independently but includes the history of the epidemic by starting from the saved state of the simulation at the beginning of each time window.

The cumulative reported cases are reproduced by the model in each time window, giving rise to a time evolution of the reproduction number *R*_*t*_ (Fig. 3a, b). The large initial value at February 28, 2020, results from a sudden increase of independent first reported infections, possibly related to people coming back from holiday. This leads to an overshoot of the *R*_*t*_ value in a strength depending on the size of the time window used for analysis (not shown). The initial estimates for *R*_*t*_ are not reliable because importation is the major contributor to the detected cases instead of local transmission events. Whereas the choice of the serial interval determines the *R*_*t*_ estimation (median values shown with black line) obtained from the EpiEstim package [7, 54], the *R*_*t*_ distribution resulting from our model is governed by literature-informed model parameters (blue curve) and detection ratio of the infected cases. Using parameters derived from fitting data from Italy yields similar results (magenta curve) (see Fig. 3a).

The nationwide NPIs imposed in Germany included the recommendation for cancelation of large events on March 10, 2020, followed by recommendation of self-isolation issued on March 12, 2020 [55]. A series of NPIs were implemented subsequently in close spacing, viz., restriction on individual movement, nationwide closure of schools and leisure-related venues on March 16, 2020, and extensive contact restrictions on March 22, 2020 [56, 57] (see Fig. 3c). The Apple mobility trend [58] observed in Germany until March 22, 2020, revealed an altered higher drift since the 7th week of the year (2020) showing a peak on February 22, 2020, and a subsequent declining trend (Additional file 1: Figure S1 [58, 59]). This was followed by a rapid decline in mobility since March 8, 2020 (Additional file 1: Figure S1). As the new cases registered were exposed 9 days earlier, the decline in the reproduction number until March 19–March 21, 2020, observed in the model is unlikely due to NPIs but can be attributed to behavioral changes. Although mobility in Germany showed an upward trend following March 22, 2020, despite extensive contact restrictions (Additional file 1: Figure S1), the reproduction number went downwards until mid-April 2020 achieving a value near unity as of April 6, 2020, in between and after a period of fluctuations, attained a minimum on May 4, 2020. In addition to demonstrating protective awareness among individuals, this illustrates that the NPIs imposed appear to have had a strong effect on the dynamics of the COVID-19 epidemic (see Fig. 3c).

NPIs were released in Germany on April 20, 2020, for the first time. Shops were opened, and a few days later, wearing masks became compulsory. The *R*_*t*_ value reacted with a delay of 15–19 days (Fig. 3c). On the 19th day following the first release, it increased by roughly 0.38, continued to be in a range of 0.66–0.75, and then decreased again by 0.15 (numbers mentioned for *μ*=0.2), presumably in response to the imposed masks. The second release of measures was widely implemented on May 11, 2020, and involved a cautious re-opening of child care and schools as well as restaurants. However, all of those were opened with imposed social distancing. Again, 19 days later, the *R*_*t*_ values increased to 0.82 as of May 30, 2020. Following a short span of slight fall in the reproduction numbers, this remained in a range of 0.80–0.88 around June 12, 2020, for Germany (Fig. 3c). This observation illustrates the sensitivity of the viral spreading to NPIs as well as the possibility to partially release NPIs without losing control of the epidemic, provided the population keeps social distancing and hygiene measures in place, and avoids inter-personal contacts [60].

While there was a large diversity of epidemic onset and intermediate developments particular to individual federal states, the overall tendency converged to values below *R*_*t*_ = 1 around the first week of April 2020 (see Additional file 1: Figure S3 [41–43]). The coherent reduction of the reproduction number after nationwide implementation of several NPIs together with further measures specifically applied in different federal states speaks for the efficiency of the measures and the responsiveness of the population to the NPIs. Re-opening and a continuously increasing trend in mobility induced resurgence of COVID-19 outbreaks in multiple federal states, such as North Rhine-Westphalia, Berlin, Brandenburg, Saxony, and Saxony-Anhalt around the second half of May and the first half of June 2020 (Fig. 3d and Additional file 1: Figure S3). This resulted in a sharp rise in reproduction numbers ranging from 2 to 2.8 in these aforementioned states around the 3rd week of June. It is also reflected in the reproduction numbers of whole Germany around that time, reaching a peak on June 20, 2020. The *R*_*t*_ values in Germany remained more than unity during June 17, 2020, to June 27, 2020, before maintaining around 0.63–0.82 until July 13, 2020, due to reimposed regional regulations in several federal states. Since then, it had increased and remained mostly in the range of 1.22–1.56 prior to displaying a downward trend since the last week of August. Such variations can be understood as an overall impact resulting from alterations in *R*_*t*_ development in individual federal states (see Additional file 1: Figure S3). One interesting observation, however, is some level of correlation between a couple of federal states with regard to the evolution of their reproduction numbers, e.g., (i) Berlin and Brandenburg and (ii) Baden-Würtemberg and Bavaria (Fig. 3d). We note that Berlin is encircled by Brandenburg whereas Baden-Würtemberg and Bavaria are neighboring federal states located at similar geographical altitude with Alps in their southern part. Such a correlation suggests that resurgence of outbreak in one region may act like a reservoir of new infections in adjacent regions.

Next, the *R*_*t*_ distribution resulting from the median values obtained in the last week in the different federal states is compared and ranked in Fig. 3e. As of August 31, 2020, most of the federal states and Germany as a country exhibited a weekly median of *R*_*t*_ around 1 or higher. Bavaria and Baden-Würtemberg which were hit early on by the COVID-19 outbreak as well as Bremen resurfaced with consistently higher reproduction numbers during the last 3 weeks of August 2020 (see Additional file 1: Figure S3). In contrast, North Rhine-Westphalia which had a significant case load during early weeks in the pandemic and also exhibited local outbreaks and super-spreading events around May–June [61] was relatively doing better in controlling the outbreak by the end of August 2020 (Fig. 3e). Among other federal states which encountered a substantial sharp increase in *R*_*t*_ at some point after re-opening (see Additional file 1: Figure S3), Mecklenburg-Western Pomerania, Berlin, Saxony-Anhalt, Brandenburg, and Hamburg showed a median *R*_*t*_ less than or around 1 whereas Saxony displayed a median *R*_*t*_ higher than 1 for the week ending on August 31, 2020 (Fig. 3e and Additional file 1: Figure S3).

The number of unregistered cases is not well known in Germany. In the model, (1−*α*)*μ* captures the registered fraction of the infected cases (Fig. 1). In order to demonstrate the importance of the number of undetected cases for the interpretation of the results, we compared the results for *μ*=1,*μ*=0.5,*μ*=0.2, and a qualitative time-varying *μ*_*t*_ informed by the mobility and testing data (see Additional file 1: Figure S1-S2 [32, 41–43, 62, 63]) for a fixed proportion of purely asymptomatic as well as unregistered individuals, i.e., *α* (*α*=0.22 unless otherwise specified). For time-invariant detection ratios [ (1−*α*)*μ*], it turns out that the *R*_*t*_ value derived from a model with more symptomatic unregistered cases (i.e., a lower *μ*) is slightly enhanced but remains in the same range, the impact being more prominent during initial weeks (Fig. 3c). Temporal evolution of *R*_*t*_ for a realistic time-varying detection ratio captures the sensitivity towards NPIs better and clearly shows the timeline of induced changes in *R*_*t*_ due to phased re-opening with a similar delay (Fig. 3c, green curve). A consistent rise in *μ*_*t*_ prior to June 2020 resulted in lower *R*_*t*_ values from April 2020 onward. Following a peak detection of infected cases around the end of May 2020, *μ*_*t*_ fell due to a lower number of tests per confirmed case compared to the increased mobility (see Additional file 1: Figure S1), causing higher *R*_*t*_ values since June 2020. The resulting reproduction numbers are not significantly sensitive to changes in *α* (see Additional file 1: Figure S4). In our model, the infectious period of individuals who remain asymptomatic throughout is assumed to be shorter than the overall infectious period (including pre-symptomatic stage) of the symptomatic people. This results in a slightly lower reproduction number as we increase *α*.

**Fig. 4:**
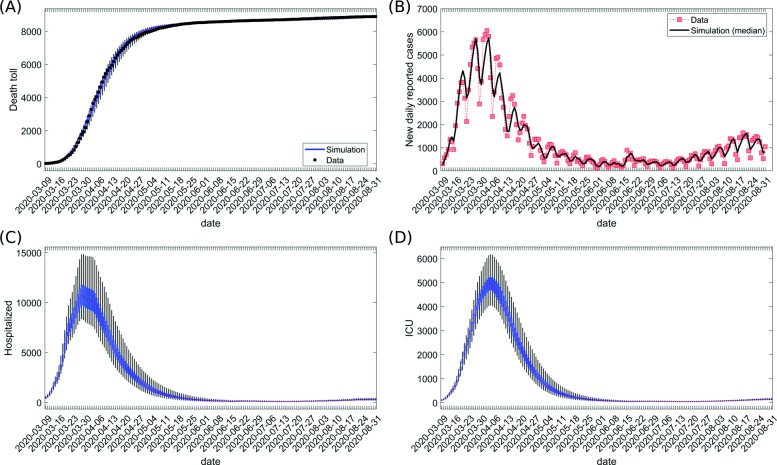
Time evolution of variables during epidemic. The distribution of simulated values for the last date of sliding time window is shown for **a** cumulative number of deaths and **b** registered daily new cases (median is shown), **c** number of hospitalized cases currently treated (census) in non-critical hospital beds, and **d** number of cases currently treated (census) in all types of critical care units (ICUs in the model). Note that the daily reported cases are calculated by subtracting the estimated cumulative number of registered cases in two consecutive dates from the sliding time windows ending at each date. The data for analysis were taken from [41–43]; own calculation and design

The model can be used to estimate the dynamics of the load for the healthcare system. Based on the resulting fitting of cumulative detected cases (Fig. 3b) and cumulative deaths (Fig. 4a, see the “Methods” section), we investigated the extent of hospitalization during the epidemic (Fig. 4 and Additional file 1: Figure S5). The number of deaths and new daily reported cases well captured the trend in the data. The highest number (median) of estimated daily reported cases was 5727 (Fig. 4b). The estimated peak (median) for healthcare usage showed 10,690 occupied hospital beds (all types of non-critical care units) on March 28, 2020, and 4938 ICU beds (all types of critical care units) on April 4, 2020 (Fig. 4c, d). These numbers stayed within the capacities of the German healthcare system [64]. The sensitivity of this result to changes in the model parameters is shown for our retrospective analysis in Additional file 1: Figure S6.

**Fig. 5:**
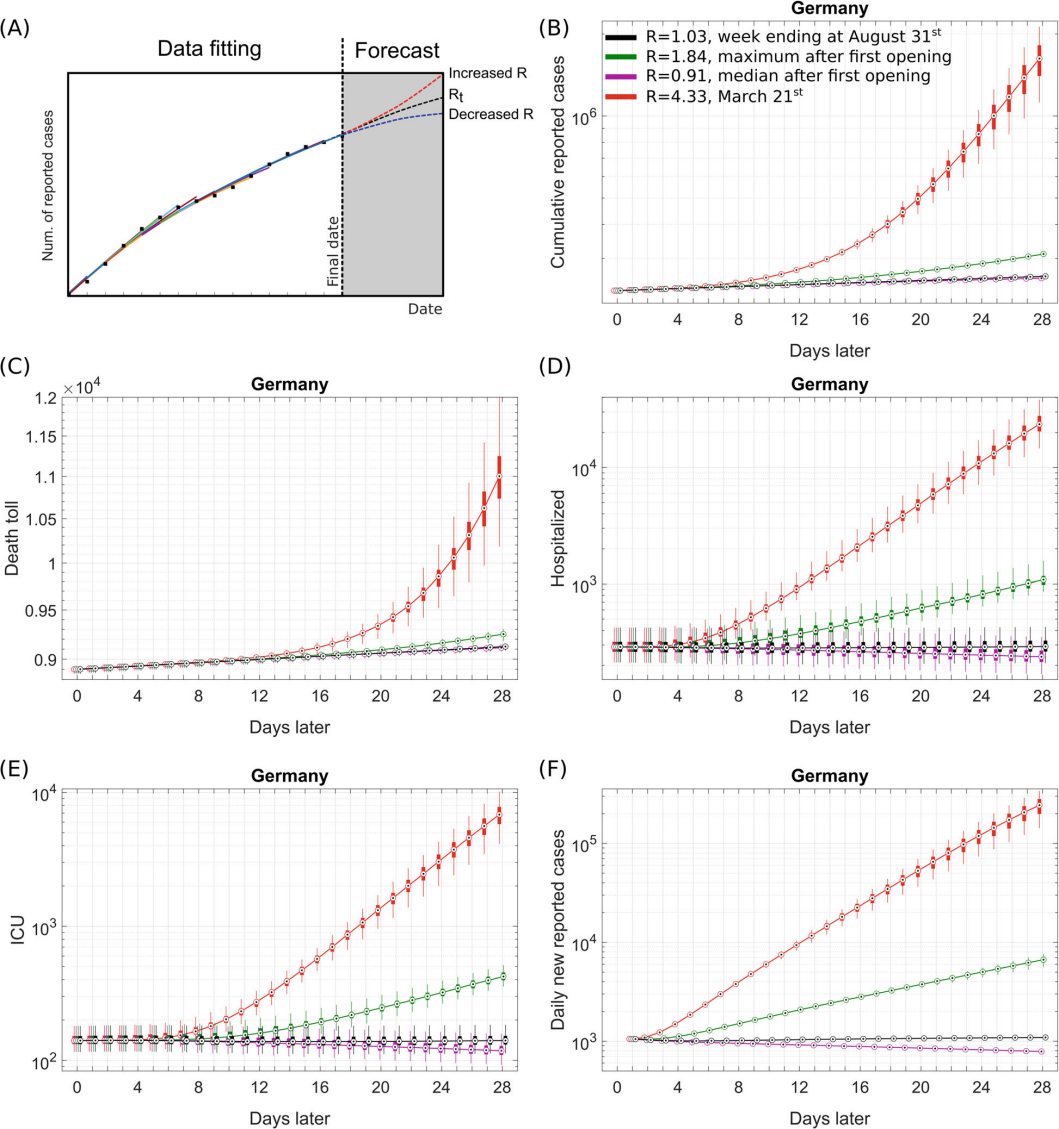
Starting from the final state in Fig. 3a, a value for the transmission rate *R*_1_ was introduced based on the *R*_*t*_ history of the pandemic and the latest hospitalization rate (as on August 31, 2020) estimated from fitting the cumulative deaths (see the “Methods” section). Results are shown for the mean of median *R*_*t*_ values observed during the last week of August 2020, i.e., *R* = 1.03 (black); the maximum *R*_*t*_ values estimated during May 5, 2020, to August 31, 2020, i.e., *R* = 1.84 (green); the median *R*_*t*_ value observed over a period from May 5, 2020, until August 31, 2020, i.e., *R* = 0.91 (magenta); and the median *R*_*t*_ value observed on March 21, 2020, i.e., *R* = 4.33. May 5, 2020, was chosen as the first re-opening started to show its first impact from this date (see the “Results” section). *α*=0.22 and *μ*=0.2 were kept fixed. The simulations were continued for 28 days from this last time point. Box plots show the 25 and 75 percentile as well as the minimum and maximum values corresponding to 100 simulations for literature-informed parameter variation (see Table 1). **a** A scheme for our projections. **b** Cumulative reported cases. **c** Cumulative deaths. **d** Hospitalized patients in non-critical care beds (census on specific days). **e** Occupied critical/intensive care units (census on specific days). **f** Daily new reported cases. All simulation results except **c** are presented on log-scale. Case data before the prospective analysis are taken from [41–43]; own calculation and design

We next investigated different prospective scenarios from the final date of the data fitting phase by retaining the state information of the model. We used the hospitalization rate estimated as on August 31, 2020 (*ρ*(*t*_latest_)), and plugged this into Eq. 19 while imposing different reproduction numbers (*R*) for the whole period of prospective analysis. *ρ*(*t*_latest_) primarily depends on the affected age groups and the extent of an effective testing-quarantine strategy (Fig. 5a). Starting from the last state of the model for Germany, thus, including the complex distribution of individuals onto the different compartments of the model at this time, the simulation was first continued for 28 days with the mean of median *R*_*t*_ values observed during the last week of August 2020, i.e., with *R* = 1.03 (base scenario). It provides a stable situation without a significant resurgence of cases in short time (Fig. 5b–f, black). The median *R*_*t*_ value observed over a period from May 5, 2020 (as the first re-opening started to show its first impact from this date), until August 31, 2020, which resulted in *R* = 0.91, contained the epidemic but was not able to stop it in a short time (Fig. 5b–f, magenta).

A release of NPIs to a degree that induces *R*_*t*_ to be the maximum value estimated during May 5, 2020, to August 31, 2020, i.e., *R* = 1.84, causes a resurgence of around 7000 daily new reported cases at the end of the 4th prospective week but does not cause a significant burden to healthcare in near future (Fig. 5b–f, green). Provided this reproduction number was kept for 1 year, around 19,500 critical care beds and around 40,000 non-critical care beds would be occupied at peak causing an overwhelmed healthcare system in comparison to around 9600 free ICUs on August 31, 2020, as per [64]. This scenario may lead to total of 80,000 deaths (see Additional file 1: Figure S7).

A relatively pessimistic prospective scenario reflecting a complete release of NPIs and the absence of any protective behavioral measure was modeled with the median *R*_*t*_ observed on March 21, 2020, i.e., with *R* = 4.33. This leads to a major immunization of the population and results in a drastic increase in healthcare usage (e.g., ≈ 7000 occupied critical care beds) and cumulative deaths (≈ 1900 higher than the base scenario) within 4 weeks (Fig. 5, red). Consequently, in the long run, it leads to an overwhelmed healthcare system (see Additional file 1: Figure S7).

**Fig. 6:**
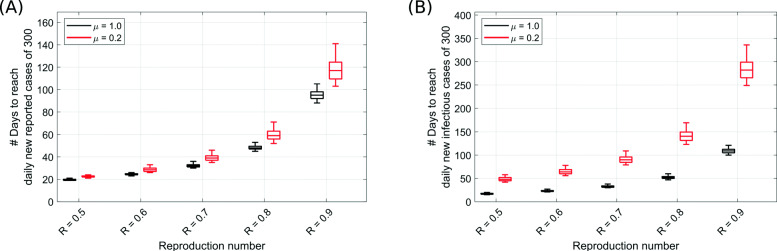
Starting from the last state of the model in Fig. 3a for Germany, the duration needed to achieve as few as 300 **a** new reported cases per day and **b** new infectious cases per day (outflow from exposed compartment, see Fig. 1) in whole Germany was estimated in a prospective analysis with different imposed fixed values of reproduction number *R*. For each setting, the results were shown for *μ*=1 (black) and *μ*=0.2 (red) to assess the impact of detection ratio ((1−*α*)*μ*) of the infected cases. *α*=0.22 and latest hospitalization rate was used. Box plots show the 75 and 25 percentile as well as the minimum and maximum values corresponding to 100 simulations for literature-informed parameter variation (see Table 1)

An important question is how long NPIs would have to be kept in place until all new cases can be controlled by public health departments. Assuming 300 detected cases per day to be manageable, we calculated the time needed to achieve this number of daily new cases given different levels of the reproduction number (Fig. 6). Given the latest reproduction number of August 31, 2020, in Germany (Fig. 6a), this number could be achieved within 2 months for registered cases, and 5 months for the infectious cases if *R*_*t*_ remains at the same level throughout (Fig. 6). The detection ratio ((1−*α*)*μ*) influences this time as can be seen for different values of *μ* (*α* = 0.22 fixed). Although the duration to reach 300 new registered cases is comparable for *μ*=1 and *μ*=0.2, the infectious population significantly increases with larger fractions of undetected infections (Fig. 6, black versus red).

## Discussion

The estimated fraction of the immunized population that we calculate as the ratio of recovered to initial susceptible population assuming some form of long-lasting immunity of infected survivors stands at the range of 0.3–3% (0.3% with *μ*=1, 1.7% with *μ*=0.2, and 2.8% for our estimated time-varying *μ*_*t*_; all with *α*=0.22 [32]) at the end of August 2020. Hence, the German population seems far from achieving herd immunity and a renewed outbreak or a second wave is extremely likely in the absence of NPIs or continued behavioral changes to prevent a viral infection. While continuation of the epidemic with *R*_*t*_ values close to unity would avoid a large number of COVID-19 infections and deaths, it may lead to a major economic burden [5], induce unemployment and related collateral damages, and increase risks of suicide in certain individuals due to lasting social distancing [65] and could pose a strong load on the healthcare system due to a delayed/neglected treatment of other diseases [66].

As expected, intensifying contact restriction measures accelerates reaching a low number of daily new cases whereas an increase in *R*_*t*_ values delays the reduction of the daily new cases. In view of the non-linearity of the delay with larger reproduction numbers, one may speculate that it might be more advantageous in terms of health and economy to maintain a lower *R*_*t*_ value less than unity through NPIs, social distancing, and protective social culture in order to quickly achieve a controllable and traceable low number of daily new cases [67]. A complete elimination of the virus, as it appears in the model, is hard to achieve in reality because of open borders to European neighbors and unknown viral properties which might allow it to reappear under particular conditions. A combined strategy of rapid identification and isolation of infection as well as efficiently traced contact clusters also worked well in Japan [68]. We showed that a large number of undetected cases increase the delay in reaching a target number of daily new cases. While seroprevalence studies suggest undetected cases substantially less than 10-fold of the detected cases in the studied communities [32, 69], the overall true number of undetected cases in Germany is still not known.

The predictive power of the model was analyzed by comparing a forecast of cumulative new reported cases for 1 or 2 weeks based on the *R*_t_ value in the past week with the corresponding cumulative increase observed in reality (Additional file 1: Figure S8). Regional outbreaks cause a sudden rise in *R*_t_ values globally, such that in this case, the prediction on the country level overestimates cumulative new cases in the upcoming days. One intriguing finding is the tendency of underestimating the cumulative new reported cases on a scale of a couple of weeks prior to a positive overshoot in prediction error (see Additional file 1: Figure S8). It can be inferred as upcoming structural increase in *R*_t_ estimations (see *R*_t_ values in June 2020, Fig. 3c). Apart from the periods of sudden rise in *R*_t_ values, the model prediction works well on a scale of a couple of weeks. Model predictions work assuming a time-invariant detection ratio in the upcoming days, which, in reality, is unlikely the case for all weeks. This would result in an overestimation when the detection ratio falls or an underestimation when the detection ratio rises. Due to having a lower chance of missing an infection induced death in the data and a longer delay to death following viral exposure, the prediction for the death toll is excellent up to 5 weeks (e.g., < 0.5% error while continuing the projection based on the fitting until August 31, 2020). It illustrates the usefulness of the model in determining the burden on the healthcare system in the near future at least on a scale of a month.

Long-term prediction for any pandemic, especially a new one, is challenging due to several less known or unpredictable factors which may impact its transmission dynamics and its effective potency. Examples of these factors include the impact of accumulation of aerosols with viable virus in closed rooms [70, 71], extent of aerosol-mediated transmission and seasonality [72, 73], alterations of behavioral response, future NPIs, and viral mutations. In addition, development of efficient rapid testing methods, extent of reinfection and inherent immunity, and improvement in clinical management would determine and modify the future course of the outbreak. Within these limitations, our model can still guide the government authorities to prepare better by projecting the peak of healthcare usage and estimates of population immunization by the pathogen as well as case fatalities under different circumstances (see Additional file 1: Figure S7).

The analysis of the individual federal states in Germany revealed local differences. The federal states appear to witness different phases of the outbreak, and NPIs exhibit different kinetics of impact. Therefore, state-specific or even district/city-specific development of *R*_t_ provides a better sense to the local authorities to plan the future course of actions to control the epidemic locally, which, if left uncontrolled, may act like a disease hot spot to initiate new clusters of infections across the federal states. One intriguing finding is that a local outbreak results in sharper changes in *R*_t_ while its global impact as observed in the country level *R*_t_ estimates is relatively damped (Fig. 3c, d, Additional file 1: Figure S3). The full analysis for *R*_t_ of all federal states is available at [11] and clearly emphasizes local heterogeneity of the epidemic. Prevalence of super-spreading events, population density, and differences in social structures can be some of the contributing factors in driving the outbreak heterogeneously across different states.

We do not use an age-stratified version of our model for the presented analysis due to incomplete age-stratified data, lack of knowledge on how the model parameters would depend on different age groups, and an undetermined and uneven testing bias across different age groups. The age-dependence is phenomenologically included in the model by using a time-dependent hospitalization rate, which reflects the demography of the infected people. With this, our goal to understand the development of time-varying reproduction number, overall usage of healthcare facilities, and future course of the outbreak can still be achieved using an age-independent mean-field approach. Even though observed case fatality ratios (and indeed infection fatality rates, too) for older age groups are much higher than for young adults and children in COVID-19 [62], we can still fit the cumulative death curve based on the estimated (informed by the literature) fraction of the hospitalized COVID-19 patients who are dying thereby enabling us to capture the time-dependent hospitalization rate eventually resulting in a time-dependent overall CFR for the reported cases.

## Conclusions

In this paper, we developed a compartmental model (SECIR) accounting for the specificities of the recent COVID-19 outbreak. We reported an adaptive methodology to estimate the time-varying reproduction number (*R*_t_) based on the incidence of reported cases. As parameterization is essential for the quality of our analysis and predictions, two reference parameter sets were determined by thorough analysis of the literature on COVID-19 and an analysis of Italian data. The results discussed are consistent between both parameter sets. Even though both parameter sets are not completely independent, this consistency increases the credibility of the model results. Implementation of NPIs in close spacing, heterogeneity in their application, and withdrawal timings across different federal states as well as cities make it statistically uncertain to disentangle the impact of a particular NPI [7]. Most importantly, the behavior of the people changes over time, examples of which include dynamic and heterogeneous compliance to NPIs and mask usage as well as behavioral exhaustion. Even though the model is constructed by taking into account the biological characteristics of the infection transmission dynamics of COVID-19 such as asymptomatic and pre-symptomatic transmission, and contribution from undetected mild-symptomatic cases, the model presented here can be easily translated to any similar infectious disease with equivalent features while such a methodology can also be applied to common infectious diseases. In addition, our results can capture the qualitative aspects of how the infection incidence, patients admitted in non-critical and critical care units, and deaths change as days progress during the COVID-19 outbreak. Furthermore, it can also guide the authorities in assessing how the pandemic would evolve in the near future and what load on the healthcare facilities to expect under certain scenarios drawn from the history of the outbreak itself. We provide a daily updated evaluation of the reproduction number suitable to support political decisions on NPIs in the course of the COVID-19 outbreak and applied to German federal state data online [11].

## Supplementary Information

**Additional file 1** Figures S1-S8, Parameter description, Additional details of Italy fitting, and Table 1.

## Data Availability

Daily updated results are provided in a version controlled repository [11] including a full revision history. The analysis is based on case data for Germany provided by the Robert-Koch-Institut as on October 27, 2020 [41]. Note that the case numbers in the data might change for newer data sets due to delayed reporting and data corrections.
