# Supplementary material for: Development of the reproduction number from coronavirus SARS-CoV-2 case data in Germany and implications for political measures
Source: BMC Med. 2021 Jan 28;19:32. doi: 10.1186/s12916-020-01884-4 (PMC7840427; doi:10.1186/s12916-020-01884-4)
Supplement: Supplementary file 1 — Additional file 1 Figures S1-S8, Parameter description, Additional details of Italy fitting, and Table 1. [file 12916_2020_1884_MOESM1_ESM.pdf]

RESEARCH

Development of the reproduction number from coronavirus SARS-CoV-2 case data in Germany and implications for political measures

Additional file 1

Sahamoddin Khailaie[1†], Tanmay Mitra[1†], Arnab Bandyopadhyay[1^], Marta Schips[1^], Pietro Mascheroni[1], Patrizio Vanella[2,3,4], Berit Lange[2,5], Sebastian C. Binder[*1] and Michael Meyer-Hermann[1,6,7*]

[*]Correspondence: sb@theoretical-biology.de | mmh@theoretical-biology.de
[1]Department of Systems Immunology and Braunschweig Integrated Centre of Systems Biology (BRICS), Helmholtz Centre for Infection Research, Rebenring 56, 38106, Braunschweig, Germany
Full list of author information is available at the end of the article
[†]These authors contributed equally to this work (alphabetical order).[^]These authors contributed equally to this work (alphabetical order).

**Figure S1:**	Apple mobility trend based on changes in routing requests and number of tests per confirmed case over time.
**Figure S2:**	Visualization of time-varying detection functions.
**Figure S3:**	Time-varying reproduction number Rt for individual federal states estimated separately.
**Figure S4:**	R_t analysis as in Fig. 3 (A) with two different values for α.
**Figure S5:**	Same analysis as in Fig. 4 but with time-varying μ_t.
**Figure S6:**	Sensitivity for the parameters that were varied for retrospective analysis where fitting is involved.
**Figure S7:**	One year projections for higher Rt -values based on the history of the pandemic.
**Figure S8:**	Prediction error for new registered cases in 7- and 14-day projections.
**Parameter description:**	Supplementary text with detailed description of parameter derivation.
**Additional details of Italy fitting:**	Supplementary text with additional information on the data fitting procedure for Italy.
**Table 1:**	Bounds used to determine the ranges of the parameters by fitting the data for different regions of Italy.

Keywords: SARS-CoV-2; COVID-19; Epidemiology; Modeling; Non-pharmaceutical interventions; Reproduction number; Healthcare usage

Mobility trend and testing data

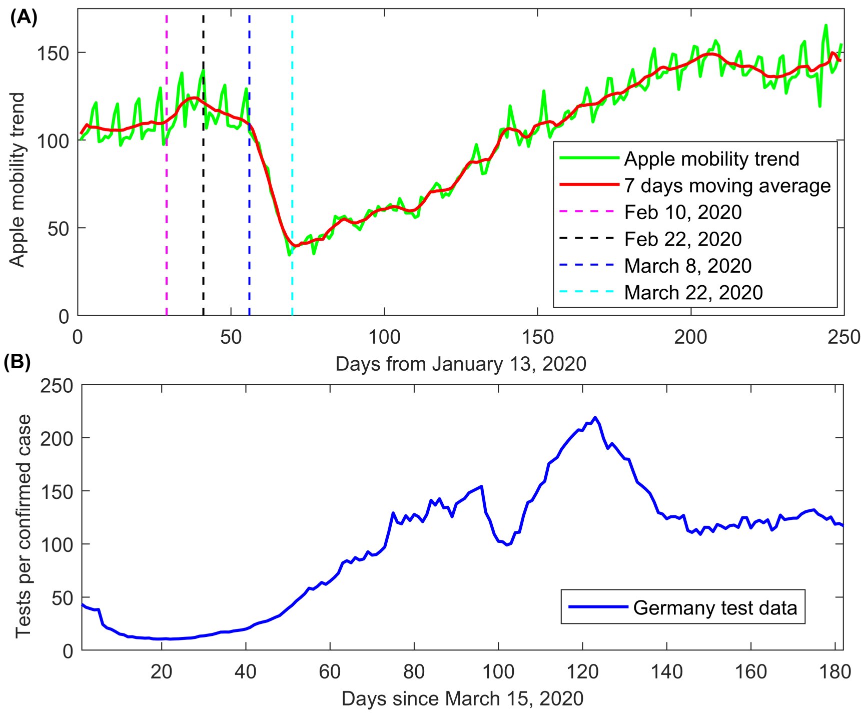

Figure S1 (A) Apple mobility trend [58] based on changes in routing requests since January 13, 2020. As per [58], the mobility was set to 100 on January 13, 2020. Equal weights has been given to walking, transit and driving data (see [58]) while calculating the overall daily mobility (green curve). As data for May 11 – May 12, 2020 is not available in their data set, we have set the mobility on these two dates by calculating the average of the entries on May 10, 2020 and May 13, 2020. In addition, the moving average of 7 days is shown (red curve) (B) Number of tests done per confirmed case. Data represents (blue curve) a moving average of 7 days and is available since March 15 [59]

Time-varying detection

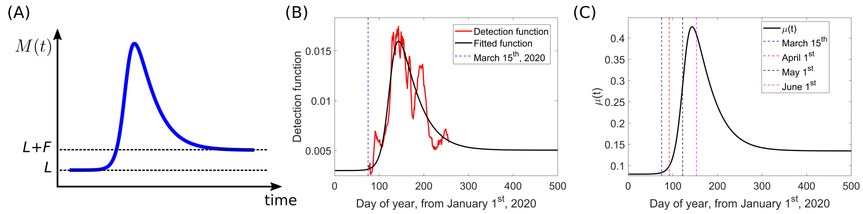

Figure S2 (A) Double sigmoidal function (formula below), (B) Double sigmoidal function is fitted to the detection function estimated from the mobility trend (7 days moving average) and number of tests per confirmed case using: detection function (t) = number of tests per confirmed case(t)/[(mobility(t − 9))^2], as in the model, the time to clinical registration from viral exposure is around 9 days. A squared mobility is taken as a proxy for inter-individual interaction. The qualitative use of a double sigmoidal function can also be understood from: (i) the testing capacity has increased with time leading to a peak in detection ratio, and (ii) the increase in mobility leads to a subsequent fall of detection ratio while the country is still testing its optimal testing capacity. (C) The resulting $\mu(t)$. μ on March 15, 2020 was estimated using an infection fatality ratio (IFR) of 0.9% adjusting the delay from symptom onset to deaths which is slightly more than 21 days in the model (as per the mean values). Basically, it assumes that the people who have died on April 3, 2020 got exposed on March 12, 2020 (the day when recommendation of self-isolation was made), i.e., on the day 22 following viral exposure. It also assumes that some of the people $[(1-\alpha)\mu]]$ among the ones who got exposed on March 12, 2020 would get registered on 21st of March as there is about 9 days delay from exposure to registration. As testing data was not found prior to March 15, 2020, the number of tests per confirmed case was assumed to have maintained in the same level as on March 15, 2020 prior to this date. This will lead a μ on March 15, 2020 by setting α ($\alpha = 0.22$ was used [32]). Please note that this IFR is used only to calculate the detection ratio on March 15, 2020 and prior to this date and hence, does not represent the overall IFR (or their temporal variation) for the analyzed course of the outbreak in Germany. The assumed IFR is consistent with an IFR calculated using demographic data for Germany and age-specific IFR reported in [62] (we assumed a lesser value considering the affected age-groups during the mentioned dates [41, 42, 43]). Assuming a higher (lower) IFR at the beginning leads to a higher (lower) value of μ on March 15, 2020 and afterwards (μ_t).

$$f_{ds}(t) = \frac{1}{[1+e^{-a_1(t-t_1)}][1+e^{-a_2(t-t_2)}]}, \tag{1}$$

$$t_{max} = \text{argmax} f_{ds}(t), \tag{2}$$

$$f_{max} = f_{ds}(t_{max}), \tag{3}$$

$$c_1 = \frac{H}{f_{max}}, \tag{4}$$

$$c_2 = \frac{H-F}{f_{max}}, \tag{5}$$

$$M(t) = L + \begin{cases} c_1 f_{base}(t) & t \le t_{max}, \\ c_2 f_{base}(t) + F & t > t_{max}. \end{cases} \tag{6}$$

The values of a_1, a_2, t_1, t_2, L, H, and F were estimated using the detection function data. The values of $M(t)$ were then rescaled by tuning the starting value of $\mu = 0.08$ (on March 15, 2020). The resulting values are $L = 0.003$, $H = 0.013$, $F = 0.002$, $a_1 = 0.1039$, $a_2 =$ -0.0277, $t_1 =$ 127, $t_2 =$ 129.5. The equations for a double-sigmoidal function are taken from [63] and here, t corresponds to day of the year starting from January 1, 2020.

R_t in individual federal states

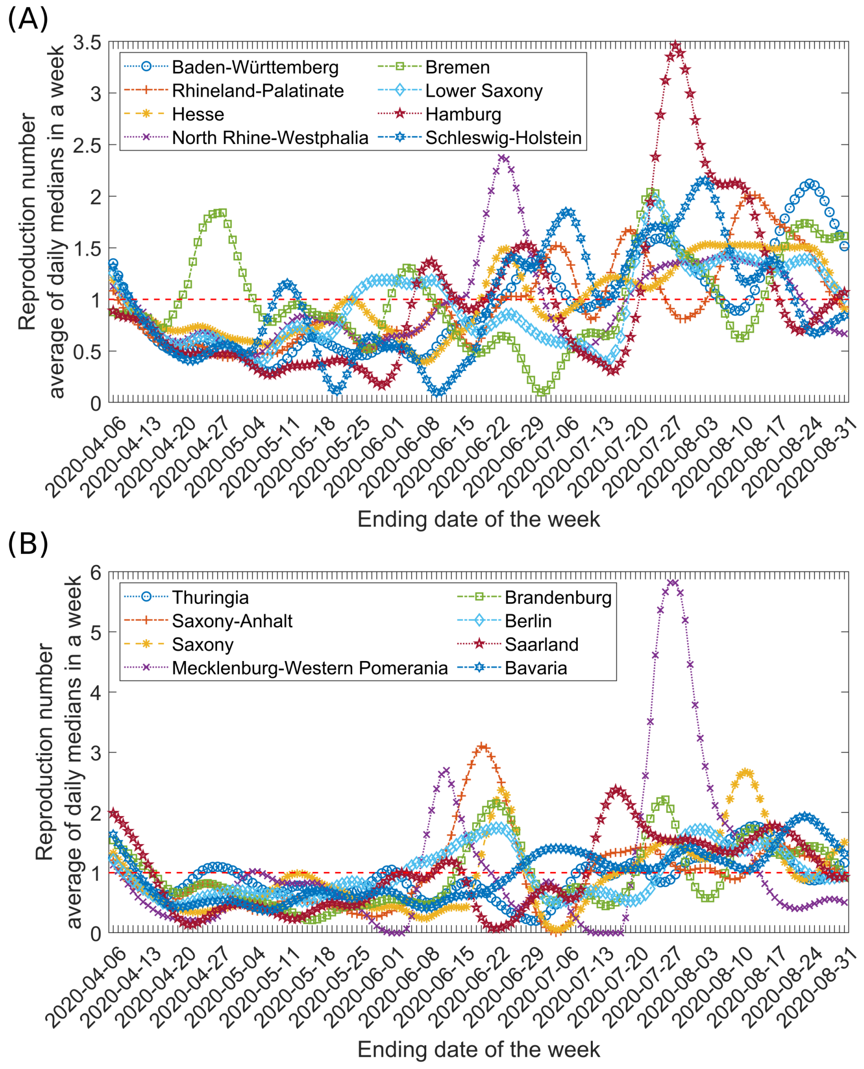

Figure S3 Time-varying reproduction number R_t for individual federal states estimated separately. The average of the daily median of R_t is calculated and shown for the ending date of the considered week. For better visualization, the results are shown in separate panels (A) and (B). Results shown for time-invariant $\mu = 0.2$ and $\alpha = 0.22$. Case data was taken from [41, 42, 43]; own calculation and design.

Sensitivity of R_t on α

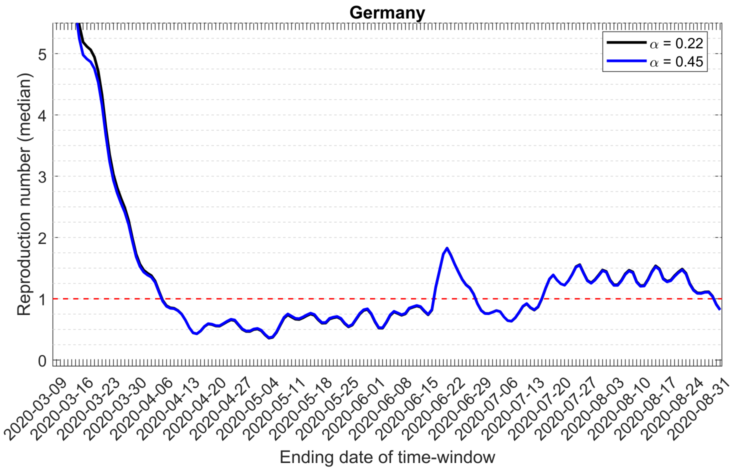

Figure S4 Same analysis of the time-varying reproduction number R_t as in Fig. 3 (A) of the main text but for two different values of parameter α. Only the R_t-values are shown. $\alpha = 0.22$ and $\mu = 0.2$ were used.

Retrospective analysis for time-varying μ_t

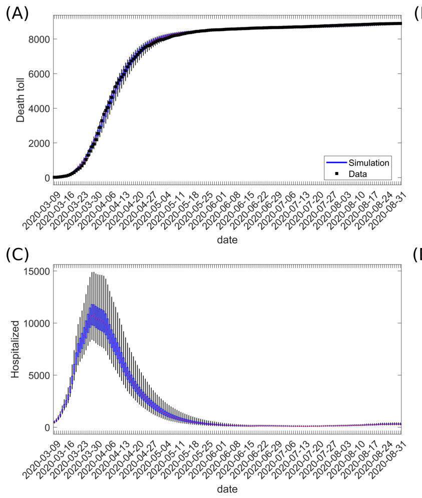

Figure S5 Same analysis as in Fig. 4 of the main text with a time-varying μ_t. $\alpha = 0.22$ is used.

Sensitivity of the varied parameters on retrospective analysis

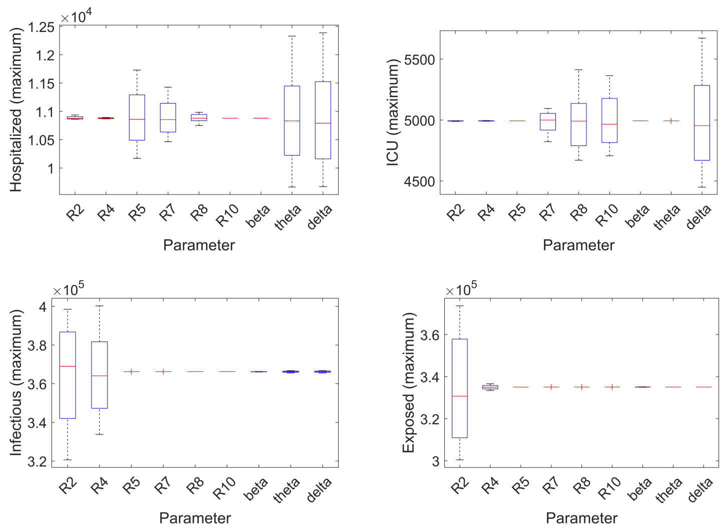

Figure S6 Sensitivity for the parameters that were varied for retrospective analysis where fitting is involved. Each parameter was varied from the range (as mentioned in Table 1 of the main text) one at a time for the analysis. $\alpha = 0.22$ and $\mu = 0.2$ were used.

Projections for one year: release of measures

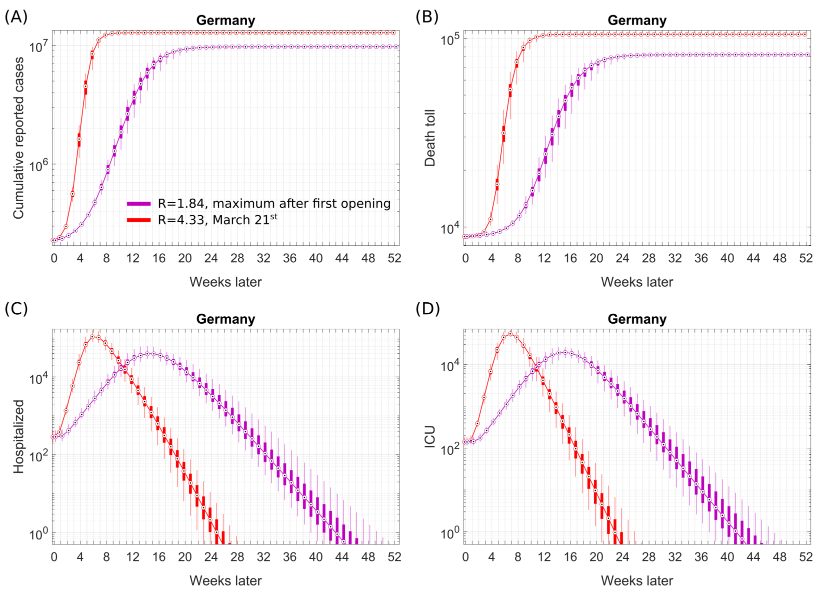

Figure S7 One year projections for higher R_t-values based on the history of the pandemic. Starting from the final state in Fig. 3 (A) (see main text), a value for the transmission rate R_1 was introduced based on the R_t-history of the pandemic and the latest hospitalization rate (as on August 31, 2020) estimated from fitting the cumulative deaths (see Methods). Results are shown for the maximum R_t-values estimated during May 5, 2020 - August 31, 2020, i.e., $R = 1.84$ (magenta) and the median R_t-value observed on March 21, 2020, i.e., $R = 4.33$ (red). May 5, 2020 was chosen as the first re-opening started to show its first impact from this date (see Results). The later of them ($R = 4.33$ (red)) results in a peak usage of around 111600 occupied non-critical care wards and around 54000 critical care units. The corresponding peak numbers for $R = 1.84$ are mentioned in the main text (see Results). The value of the peaks increases with increasing the release of the measures, and the time of the peak occurs earlier. $\alpha = 0.22$ and $\mu = 0.2$ were kept fixed.

Prospective projection error

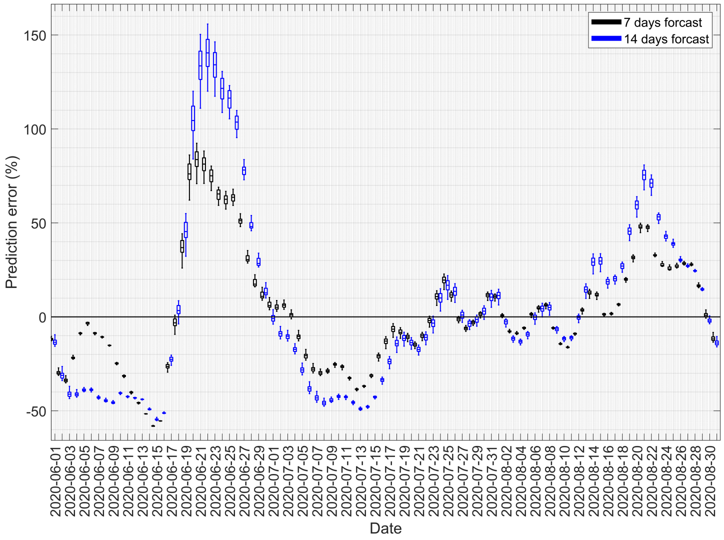

Figure S8 Prediction error for new registered cases. We measure the increase in case numbers following a particular date for the next 7 days or 14 days and depict the corresponding error at that particular date comparing it with the increase in case numbers as per the data. See discussions on this in the main text.

Parameter description:

R_1: R_1 represents the product of median contact frequency for a population and the transmission probability of COVID-19 per each contact made with an infectious person (C_I, C_R, I_X, I, I_R and I_H in the model).

R_2 and R_3: To estimate R_2, one needs to know the duration for which an individual remains in a latent non-infectious stage following the transmission of COVID-19 (inverse of this gives R_2), whereas R_3 can be estimated as the inverse of the pre-symptomatic infectious period. The pre-symptomatic infectious period ($\frac{1}{R_3}$) is estimated to be around 2 days in literature [21, 22]. With a mean incubation period ($\frac{1}{R_2} + \frac{1}{R_3}$) of 5.2 days [8], the mean latent non-infectious period following viral exposure ($\frac{1}{R_2}$) turns out to be 3.2 days. In an optimistic scenario where symptomatic individuals are immediately and properly quarantined (i.e., with no infection transmission afterwards) following symptom onset, if it is assumed that subsequent infections can occur at random during the pre-symptomatic infectious period only, the mean of shortest (assuming no restriction prior to symptom onset) serial interval will be the sum of the average latent period (from infection to infectiousness) and half the average infectious period before disease onset (i.e., $\frac{1}{R_2} + 0.5\frac{1}{R_3}$). This results in 4.2 days which is closer to the shorter estimates of mean serial interval [6, 12].

R_4, R_{11} and R_{12}: The inverse of R_4 is the duration for which the infected individuals with mild symptoms and not requiring hospitalization (including the unregistered symptomatic people, i.e., I_X), remain infectious after their symptom onset. To estimate this, we have made use of one study with nine young patients with no underlying health conditions, where the excretion dynamics of reproductive viruses [23] from samples of the throat and sputum were examined. This study suggests active virus replication in the upper respiratory tract in the earlier phase of the disease following onset of symptoms. RT-PCR tests result in detectable viral subgenomic messenger RNAs (sgRNA) in swabs from throat in the first 5 days after symptoms onset (In Figure 1 (d) in [23], the throat swab cultures are positive up to the 4th day, which the authors mark as sample of 4/5 days). However, we note that active virus is found in the sputum until day 8 for these mildly ill cases. In another study, it was shown that infectivity might be low from the ninth day after symptom onset [25]. Based on these studies, we assume that the mean infectivity period ($\frac{1}{R_4}$) of the mildly symptomatic individuals is 7 days, which is also consistent with an estimated infectivity period found elsewhere [24]. Please note, in the model,

$$\frac{1}{R_{11}} + \frac{1}{R_{12}} = \frac{1}{R_4}, \tag{7}$$

where $\frac{1}{R_{11}}$ represents the mean duration spent with symptoms prior to clinical registration (i.e., in state I) and $\frac{1}{R_{12}}$ denotes the remaining mean infectivity period of the registered mildly symptomatic people not requiring hospitalization (i.e., I_R). The delay in clinical registration ($\frac{1}{R_{11}}$) is estimated around 3.7 days for Europe [30]. Hence, $\frac{1}{R_{12}}$ can be easily estimated using the relation $\frac{1}{R_{11}} + \frac{1}{R_{12}} = \frac{1}{R_4}$.

R_5: The inverse of R_5 depicts the mean duration for which the hospitalized patients not requiring further critical/intensive care remain under general hospital care before getting discharge. $\frac{1}{R_5}$ depends on the age structure of the population in consideration. It also depends on the specific protocols that have to be satisfied prior to discharge. In the earlier days of the pandemic in China, often a negative RT-PCR test was set as a criteria for discharge, resulting in 10 days [13] to 14 days [14] of hospitalization even for the mild cases. We have set 8 days as the mean value for $\frac{1}{R_5}$ based on the ISARIC report of July 13, 2020 [27]. This is also consistent with the estimate mentioned in [26]. Please note that the time spent in a non-critical ward (H_S in this specific context) following the shifting of the patient from a critical care unit (U_R to H_S), is also taken as $\frac{1}{R_5}$ in the model. This is consistent with the estimated mean duration of about three to four weeks [14] that a critically ill survivor spends in the hospital in total. See the discussion on R_8 as well.

R_{11} and R_6: In the model, $\frac{1}{R_{11}} + \frac{1}{R_6}$ denotes the time a patient spends at home before hospital admission due to worsening of the disease condition. We assume that the patients are admitted to the hospital following the onset pneumonia and/or shortness of breath. One Chinese case series [15] reports a median duration of 4 days as the time span that leads to pneumonia in case of COVID-19 following manifestation of disease symptoms. Another study [13] finds the median duration from onset of symptoms to onset of breathing difficulty to be 5 days. A third Chinese case series [16] based on 298 patients admitted to one hospital in Shenzhen has reported that the median time span from disease onset to hospital admission was 5 days. Based on a German literature [28], we have estimated the mean of this duration to be around 4.25 days using the sample size, median and interquartile range reported in the mentioned study with the formulation provided in [17].

R_7: Inverse of R_7 represents the time span spent following hospitalization to admission in an intensive or a critical care unit either due to acute respiratory distress syndrome (ARDS) or other critical health issues resulting in relation to the infection. The German literature mentioned in the previous paragraph [28] also reports the time to admission in critical care units for such patients after symptom onset (i.e., $\frac{1}{R_{11}} + \frac{1}{R_6} + \frac{1}{R_7}$ in our model). From there, we first estimated the mean of this duration to be around 8.5 days using the sample size, median and interquartile range reported in [28] with the formulation provided in [17]. As the mean of $\frac{1}{R_{11}} + \frac{1}{R_6}$ is 4.25 days as discussed earlier, it results in a mean value of 4.25 days for $\frac{1}{R_7}$. In the case of Italy, the mean $\frac{1}{R_7}$ was estimated to be around 1 day using the ISARIC report of July 13, 2020 [27]. This estimate for Italy turns out to be similar to a Chinese case series [13].

R_8: The inverse of R_8 depicts the time span spent in a critical care unit before getting shifted to a non-critical care unit again following improvement in health status. We estimated $\frac{1}{R_8}$ to have a mean value around 9 days based on information provided by [26] by adjusting for the heterogeneity of stay observed for ventilated and non-ventilated critically ill patients according to the share of such cases.

R_9: The inverse of R_9 is the duration for which the asymptomatic infected individuals remain infectious following their latent non-infectious period. As these individuals do not show symptoms, we assume that they remain infectious for a shorter time as compared to those who develop even milder symptoms. From the aforementioned discussion, we note that the cases with mild symptoms remain infectious for a period of $\frac{1}{R3} + \frac{1}{R4}$. Hence, our assumption restricts $\frac{1}{R9} < \frac{1}{R3} + \frac{1}{R4}$. If we further assume that asymptomatic people are following a similar trajectory as the people with mild symptoms, and randomly become non-infectious during the whole duration of $\frac{1}{R4}$, this would result in a mean value of $\frac{1}{R9} \sim \frac{1}{R3} + \frac{1}{2\,R4}$. Therefore, mean R_9 has to be recalculated based on this formulation whenever there is a change in $\frac{1}{R3}$ or $\frac{1}{R4}$.

R_{10}: The inverse of R_{10} denotes the time span a patient admitted in ICU spends there before dying. It is estimated from time to death from onset of symptoms, which is reported to be around 16 days (i.e., on day 22 after viral exposure) [29]. Hence, the mean value of $\frac{1}{R_{10}}$ can be calculated using the following:

$$\text{Time to death from onset of symptoms} = \frac{1}{R_{11}} + \frac{1}{R_6} + \frac{1}{R_7} + \frac{1}{R_{10}} \tag{8}$$

This gives: $\frac{1}{R_{10}} \sim 7.43$ days as an average estimate for Germany.

α: This fraction represents purely asymptomatic people who remain unregistered. We can also have an idea about this fraction from the manifestation index [26]. The manifestation index describes the proportion of those infected who actually fall ill. Three studies from different settings (cruise ship outbreak, evacuated returning travellers, contact-based case search) gave figures of 51% [18], 69% [19] and 81% [20]. For the presented results specific to Germany, it was set to 22% as inferred from [32] (unless otherwise mentioned). However, we have also provided the robustness of our results with $\alpha = 45\%$ (see SI figure, alpha sensitivity). While doing the analysis for Italy, $\alpha = 42.5\%$ was used as estimated from [31].

β: It represents the risk of infection from the registered and quarantined ($I_R + I_H$) patients and, hence, captures the risk from those who are not yet effectively isolated. For Europe, we assumed it in the range of 0.05 - 0.25.

ϑ (Fraction $\frac{U}{H}$): In our model, the compartment representing ICUs include all types of critical care units (low ICUs, high ICUs, ECMOs, HDUs, CCUs and other critical care beds if any). While estimating the fraction that would need critical care, ϑ, one needs to consider that a patient can die only from the compartment U_D as per the model construction. We estimated the range of 42% to 53% for ϑ in Germany assuming the propensity of deaths to be higher among the ventilated ICU patients (ICUs as per our definition) than that among the non-ventilated ones [33, 34].

***δ** (Fraction $\frac{D}{U}$):* In an ideal healthcare system where we have enough supply of resources (e.g. ICUs, hospital beds), it can be assumed that patients only die after being admitted in critical/intensive care units. Derivation from [33] results in a ϑ_{min} of 42%, which we can then use to estimate δ_{max} (53%) using $\vartheta\delta \sim 22\%$ as shown in [33]. Assuming a lower death rate from critical care units(δ_{min}) of 42% (see relevant estimates in [34]), ϑ_{max} of around 53% can be estimated. See the section on ϑ for inherent assumptions.

***ρ** (Fraction $\frac{H}{I}$:* It might be difficult to calculate from the earlier Chinese case studies because even people with non-severe courses of disease were admitted to hospitals for isolation [26]. Based on the estimated ranges of ϑ and δ in Germany (see below), we estimated $\rho(t)$ over time by optimizing the fit of the model results to the case fatality data (see Methods) until August 31, 2020. Fitting to the Italian data was performed in a single stretch until March 18, 2020 using data for registered and quarantined cases ($I_R + I_H$), patients hospitalized in wards ($H_R + H_U + H_S$), admitted in critical care units ($U_R + U_D$), and dead (D) without incorporating any time-varying parameter (see Methods). Hence, ρ, ϑ and δ were determined by the optimization process itself. During the optimization of the fit, it was restricted that deaths occur on day 22 following the viral exposure, resulting in a constrained search of $\frac{1}{R_6}$ and $\frac{1}{R_{10}}$ (as the incubation period and Europe specific registration delay and $\frac{1}{R_7}$ were already estimated).

Additional details of Italy fitting

See the methods sections and the subsection on parametrization. The procedure is performed for Italy and for each Italian region where the first registered case is no later than February 28, 2020. Steps are mentioned below:

- Infection fatality ratio = 1.7% [35, 36];
- Total detectable cases (detected + undetected) on March 18, 2020: (dead at March 31, 2020)/IFR;
- Detection ratio, i.e., $(1-\alpha)\mu$: (infected detected)/(total detectable cases) on March 18, 2020;
- Number of detectable cases at day t: (new cases at day t)/(detection ratio); this was used to set initial conditions (see methods).
- μ = (detection ratio)/$(1-\alpha)$.

The simulation starts 8.9 days (incubation period + registration delay) prior the first registered case. For simulation details, see methods section.

Tables

Table 1 Bounds used to determine the ranges of the parameters by fitting the data for different regions of Italy.

Parameter	Bound MIN	Bound MAX	Remarks
α	0.425	0.425	Fixed [31]
β	0.05	0.25	Assumed
ρ	0.01	0.9	Varied in a broad range
ϑ	0.01	0.7	Varied in a broad range
δ	0.3	0.9	Varied in a broad range
μ	NA	NA	Calculated for each region, varies 0.0613–0.1992 across regions
R_1	0	3	Varied in a broad range
R_2	0.2188	0.4062	30% around estimated mean
R_4	0.1	0.1857	30% around estimated mean
R_5	0.0875	0.1625	30% around estimated mean
R_6	0.2906	6.8421	Note, actually inverse of $(1/R_6 + 1/R_{11})$ was varied within 0.14 – 0.26
R_7	1	1	Fixed [27]
R_8	0.0778	0.1444	30% around estimated mean
R_{10}	0.0897	0.1273	Resulting from constraints. Note, $1/R_6 + 1/R_{11} + 1/R_7 + 1/R_{10} = 16$ days
R_{11}	0.2703	0.2703	Fixed [30]
